# Regional Analgesia in Video-Assisted Thoracic Surgery: A Bayesian Network Meta-Analysis

**DOI:** 10.3389/fmed.2022.842332

**Published:** 2022-04-06

**Authors:** Jingfang Lin, Yanling Liao, Cansheng Gong, Lizhu Yu, Fei Gao, Jing Yu, Jianghu Chen, Xiaohui Chen, Ting Zheng, Xiaochun Zheng

**Affiliations:** Department of Anesthesiology, Fujian Provincial Hospital, Shengli Clinical Medical College of Fujian Medical University, Fuzhou, China

**Keywords:** regional analgesia, post-operative pain, Bayesian network meta-analysis, element analysis of Bayesian network, video-assisted, thoracic surgery

## Abstract

**Background:**

A variety of regional analgesia methods are used during video-assisted thoracic surgery (VATS). Our network meta-analysis (NMA) sought to evaluate the advantages of various methods of localized postoperative pain management in VATS patients.

**Methods:**

PubMed, the Cochrane Library, and EMBASE were searched from their date of inception to May 2021 for randomized controlled trials (RCTs) comparing two or more types of locoregional analgesia in adults using any standardized clinical criteria. This was done using Bayesian NMA.

**Results:**

A total of 3,563 studies were initially identified, and 16 RCTs with a total of 1,144 participants were ultimately included. These studies, which spanned the years 2014 to 2021 and included data from eight different countries, presented new information. There were a variety of regional analgesia techniques used, and in terms of analgesic effect, thoracic epidural anesthesia (TEA) [SMD (standard mean difference) = 1.12, CrI (Credible interval): (−0.08 to −2.33)], thoracic paravertebral block (TPVB) (SMD = 0.67, CrI: (−0.25 to 1.60) and erector spinae plane block (ESPB) (SMD = 0.34, CrI: (−0.5 to 1.17) were better than other regional analgesia methods.

**Conclusion:**

Overall, these findings show that TEA, TPVB and ESPB may be effective forms of regional analgesia in VATS. This research could be a valuable resource for future efforts regarding the use of thoracic regional analgesia and enhanced recovery after surgery.

**Systematic Review Registration:**

Identifier [PROSPERO CRD42021253218].

## Introduction

Thoracotomy is one of the most painful surgical operations performed (1); up to 65% of patients acquire chronic post thoracotomy pain syndrome (PTPS), and 10% suffer life-altering, debilitating pain (2). The most effective forms of thoracotomy are minimally invasive. According to the Lancet, video-assisted thoracic surgery (VATS) has become more common over the past decade (3). Compared with open thoracotomy, VATS reduced postoperative pain, morbidity, and length of stay (LOS) (4, 5). Nevertheless, VATS still causes moderate to severe postoperative pain and a high risk of chronic postsurgical pain (P) (6). The major cause of the majority of thoracoscopic discomfort is the intercostal incision, which cuts through the skin, subcutaneous tissue, muscle layers (including the intercostal muscles, latissimus dorsi, serratus anterior, and pectoralis major), and the parietal pleura (7). As said, intercostal nerves innervate the skin, subcutaneous tissues, and intercostal muscles. The thoracodorsal and the long thoracic nerves supply the latissimus dorsi and serratus anterior muscles. The intercostal nerves and the phrenic nerve both contribute to the parietal pleura (8).

Locoregional analgesia, aided by advances in precise and flexible anesthetic methods, has the potential to reduce postoperative pain and the occurrence of CPSP (9) and enhance early recovery after surgery (ERAS) (10); therefore, it is important to optimize post-VATS analgesia.

Thoracic epidural anesthesia (TEA) and thoracic paravertebral block (TPVB) are the gold standards for pain treatment during open thoracotomy (11). Using TEA and TPVB reduces the sympathetic reaction to surgery and improves coagulation and endocrine and immunological function (12). The issue of whether TEA and TPVB are also the gold standard in VATS has been raised (13, 14). Furthermore, adverse effects and the costs of TEA (9) raise the risk of dural puncture, nerve damage, epidural hemorrhage, and hypotension (15). The fact that minimally invasive surgery might require less-invasive analgesia has been shown (13).

With the advent of ultrasonography, several studies have described different analgesic procedures for VATS, SAPB (16), ESPB (17) and other ultrasound technologies that may provide comparable analgesia for post-VATS pain. Several randomized controlled trials (RCTs) and paired meta-analyses using only the direct comparison model have been conducted to gather reliable evidence on the best analgesic strategy for VATS. Some studies argue that TEA is unnecessary during lobectomy *via* VATS since it causes vomiting, hypotension, pruritus, and other side effects (13, 18). Another study corroborates that the gold standard is still TEA or PVB (19). Furthermore, according to some articles, ESPB is not a good therapy for pain following VATS (20). The agreement has not been confirmed. To identify the best available therapies, network meta-analysis, which synthesizes information from direct and indirect comparisons, is required.

We set out to test the above hypothesis by performing a Bayesian network meta-analysis (NMA), which enables synthesis of all direct and indirect evidence and allows the comparison of multiple treatments simultaneously within a single analysis (21). Thus, the main purpose of our study was to identify the optimal locoregional analgesia for pain management after VATS by summarizing and analyzing the available evidence.

## Methods

This network meta-analysis was performed following the Preferred Reporting Items for Systematic Reviews and Meta-Analyses (PRISMA) extension statement (22), according to the Cochrane Handbook for Systematic Reviews of Interventions (23). The protocol was registered in the Prospective Register of Systematic Reviews (PROSPERO CRD42021253218) because all analyses were based on previously published studies, and no ethical approval or patient permission was necessary.

### Data Sources and Search Strategy

PubMed, the Cochrane Library, and EMBASE were searched from their date of inception to May 1, 2021, for RCTs comparing two or more types of locoregional analgesia in adults using any standardized clinical criteria (23). Medical Subject Heading (MeSH) and text terms were combined and followed by Boolean logical operators. The language was limited to English, and an exhaustive search was conducted by using the following MeSH terms: “thoracic surgery, video-assisted,” “epidural block,” “paravertebral block,” “serratus anterior plane block,” “erector spinae plane block,” “intercostal nerve block,” and additional relevant conceptual keywords. The detailed search strategy is presented in Supplementary File 1.

In addition, we manually searched the reference lists of relevant reviews and the proceedings from major international conferences and medical journals to avoid missing any potential eligible trials. We processed the records from the above screening by using Endnote X8 (Thompson ISI Research Soft, Philadelphia, PA, United States) literature management software. Three investigators (J-FL, Y-LL and C-SG) independently reviewed all titles and abstracts for relevance. Disputes were settled by consensus and arbitration by a panel of experts. If the data were insufficient, the entire text was requested so the authors could assess the study for eligibility.

### Inclusion and Exclusion Criteria

In accordance with the PICOS selection criteria, we used the following inclusion and exclusion criteria: (1) Participants: all patients ≥ 18 years of age undergoing VATS with general anesthesia. (2) Interventions: patients receiving TEA, TPVB, SABP ESPB or ICNB as postoperative analgesia. (3) Comparisons: the control group had another type of thoracic regional block. (4) Outcomes: pain scores [visual analog scale (VAS), visual rating scale (VRS) or numeric rating scale (NRS) score 24 h postoperatively]. (5) Study design: RCTs were eligible regardless of publication year, and language limited to English.

The exclusion criteria were as follows: (1) Participants: patients who refused to receive postoperative analgesia. (2) Interventions: there was only one type of nerve block in the article or the definition of nerve block in the study was vague. (3) Comparisons: the control group did not receive any nerve block. (4) Outcomes: clinical record data were not available. (5) Study design: non-randomized controlled studies, such as case–control studies, cohort studies, and full texts, but unpublished.

### Outcome Measurement and Quality Appraisal

According to the Cochrane Handbook-designed table, we first analyzed the included studies. Four authors (JL, LY, FG, and JY) extracted relevant data from the studies. Basic items were extracted: the first author, year of publication, number of patients, detailed intervention protocols, and type of pain score. The primary outcomes were the static pain scores, and secondary outcome measures included dynamic pain scores.

The risk of bias (ROB) assessment was performed in Reviewer Manager (5.2 version). Included articles with clear explanations of random sequence generation and allocation concealment have a low risk of bias, whereas those without explanations have a high risk (23). We evaluated performance and detection bias in terms of blinded participants, staff, and outcome assessors. We consider studies at risk for attrition bias if key data, especially primary outcome data, were lacking. We evaluated selective bias based on secondary outcomes or insufficient data, such as participant characteristics. A less rigorous research design and apparent disparity when compared to previous studies are examples of less rigorous research designs that may have contributed to bias in their results. A study’s inability to address important problems evident in all of the aspects was determined to have an “unclear risk of bias.” Discussion with or impartial judgment by a seasoned expert was used to address the issue.

### Statistical Analysis

The Bayesian network approach outperforms conventional meta-analysis since it synthesizes data from many studies at once. This gives researchers more freedom to employ complex models and discern causal relationships with more scientific rigor (24). Using a random effect Bayesian statistical model and a connected network of direct and indirect data, we evaluated five types of regional analgesia simultaneously.

We started with a traditional pairwise meta-analysis of all available comparisons for each contrast. Given that our outcomes are continuous variables, the effect size of the locoregional analgesia interventions was assessed with the standardized mean difference (SMD) and 95% credibility interval (CrI). We used an adjusted comparison funnel plot to detect the presence of bias, such as publication bias and selective reporting. Network transitivity, the most important underlying assumption in NMA, had a direct effect on our study (25). To guarantee that different treatment comparisons were sufficiently comparable to give valid indirect inferences, we verified the transitivity assumption by comparing clinical and methodological features, such as patients and experimental designs, across all studies included (23). We utilized the “node-splitting” method to see whether a possible source inconsistency was present in our network (26) by analyzing direct and indirect evidence throughout the network (with a *p* value higher than 0.05 indicating consistency generation) (27).

Briefly, a network diagram was created to show all of the available evidence for each therapy. STATA (version 14.0, Corp., College Station, TX, United States) was used to carry out the aforementioned series of analyses. If the data we wanted to extract for our analysis (such as the mean, SD or sample size) were not provided in the article, we represented them by computing other accessible values, such standard errors, confidence intervals, or other statistical indices as described above, that may help to explain the SD (28). Under the Bayesian framework, restricted maximum likelihood was used to estimate parameters. In OpenBUGS (version 3.2.3), network meta-analyses for optimum locoregional analgesia for pain management following VATS were carried out in a Bayesian framework utilizing the Markov chain Monte Carlo simulation method. Because the majority of direct evidence came from a single experiment, a random fixed effects consistency model was utilized. To fit the model, we employed non-informative uniform and normal prior distributions, as well as three alternative sets of starting values (29). To simulate an accurate estimate for statistical modes, three parallel Markov chains were built with a randomly selected state (30). To minimize autocorrelation, 50,000 data points were added to both sample iterations and burn-ins, and the thinning interval was increased to 10 to generate 2,000 sample iterations with 20,000 burn-ins and a thinning interval of 1. For the objective response rate and toxic effects, 50,000 data points were added to both the sample iterations and burn-ins, and the thinning interval was increased to 10 (30, 31). The network meta-analysis evaluated the overall ranks of therapies for pain management using a Bayesian framework. A simple numerical statistic cumulative ranking probability plot was used to describe the surface under the cumulative ranking curve (SUCRA) for each treatment. A higher SUCRA score implies a better probability of a particular therapy being in the top tier or very successful, while a value of zero indicates that the treatment is unquestionably the worst (32).

## Results

### Baseline Characteristics and Risk of Bias Quality Evaluation

**FIGURE 1:**
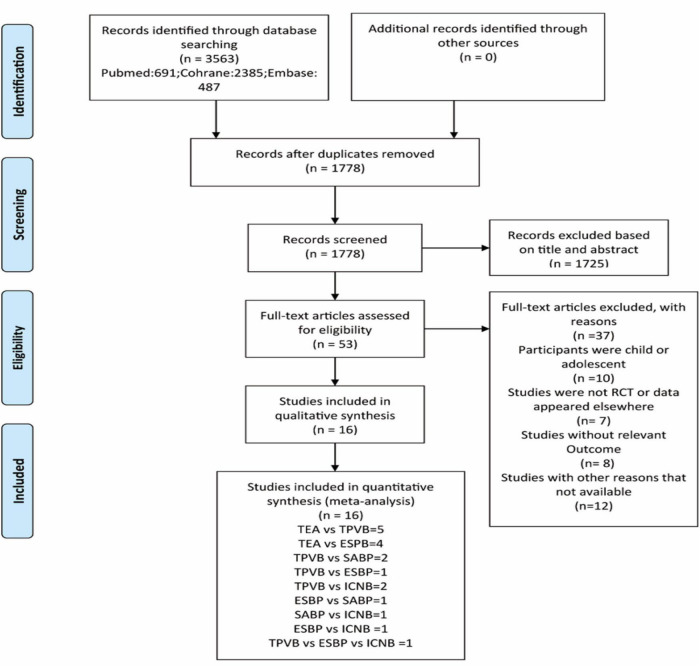
Flow chart. TEA epidural block; TPVB paravertebral block; SABP serratus anterior plane block; ESPB erector spinae plane; ICNB intercostal nerve block; RCT, Randomized controlled trials. PRISMA flow diagram for the literature search with reasons for exclusion.

A total of 3,563 records were obtained from the initial literature search. Title and abstract screening identified 53 potentially eligible articles. Based on the full-text examination, 37 records were excluded for various reasons: 10 studies had not enrolled adults, 7 studies were not RCTs, 8 studies reported data that could not be extracted, 12 studies included combined therapies sequentially or with an ambiguous definition of therapy, and 2 studies did not report the relevant outcome measure. In summary, only 16 publications (6, 14, 33–46) were deemed eligible and included in our final NMA. These trials, which spanned the years 2014 to 2021 and contained data from eight different countries, involved 1,144 people and covered five different types of locoregional analgesia for pain management after VATS. Figure 1 depicts the process of literature selection. The major characteristics of participants and treatments for the 16 trials are described in Table 1. All patients were over the age of 18 and had undergone thoracoscopic surgery.

**TABLE 1:** Characteristics of the included studies.

Study	Country	Interventions group/Control group	Sample size	Age	Scoring criteria	Major outcome
Okajima et al. (33)	Japan	TPVB/TEA	36/33	18–75	VRS	Static pain
Sylweriusz et al. (63)	Poland	TPVB/TEA	26/25	18–85	VAS	Static pain Dynamic pain
Ding et al. (35)	China	TPVB/TEA	70/32	18–80	VRS	Static pain Dynamic pain
Yeap et al. (14)	Italy	TPVB/TEA	80/40	>18	VAS	Static pain Dynamic pain
Huang et al. (36)	China	TPVB/TEA	77/39	18–80	NRS	Static pain
Merve, (64)	Korean	TPVB/SAPB	31/31	18–65	VAS	Static pain Dynamic pain
Turhan et al. (38)	Turkey	TPVB/ESPB/ICNB	35/35/36	>18	VAS	Static pain Dynamic pain
Hutchins et al. (39)	United States	TPVB/ICNB	23/25	>18	NRS	Static pain
Qiu et al. (40)	China	TPVB/SAPB	30/30	18–70	VAS	Static pain Dynamic pain
Xiang et al. (41)	China	TPVB/ICNB	40/40	18–20	NRS	Static pain Dynamic pain
Taketa et al. (42)	Japan	TPVB/ESPB	41/41	20–80	NRS	Static pain Dynamic pain
Fu et al. (43)	China	TPVB/ESPB	22/20	18–80	VAS	Static pain Dynamic pain
Dylan, (65)	United States	ESBP/SABP	30/30	18–80	VRS	Static pain Dynamic pain
Horth et al. (44)	Canada	ESBP/ICNB	12/12	>18	NRS	Static pain Dynamic pain
Lee et al. (46)	Korea	SABP/ICNB	25/25	18–80	NRS	Static pain
Chen et al. (45)	China	ESBP/ICNB/TPVB	24/24/24	18–75	VAS	Static pain Dynamic pain
						

The individual and overall study-level quality are plotted in Supplementary Figure 1. All 16 included trials reported adequate random sequence generation, 9 RCTs described their allocation concealment approach, 15 RCTs had low bias with regards to both performance and detection items, 1 study had a high risk of performance bias, and 1 study had a high risk of detection bias. One RCT had a high risk of bias with regard to attrition. The values reflected that our preliminary meta-analysis demonstrated mild heterogeneity among the included studies (*I*^2^ = 10.9%, *p* = 0.09). The funnel plot indicates publication bias, given the scatter on the inverted funnel plot (Supplementary Figure 2).

### Pairwise Meta-Analysis and Network Meta-Analysis Results

**FIGURE 2:**
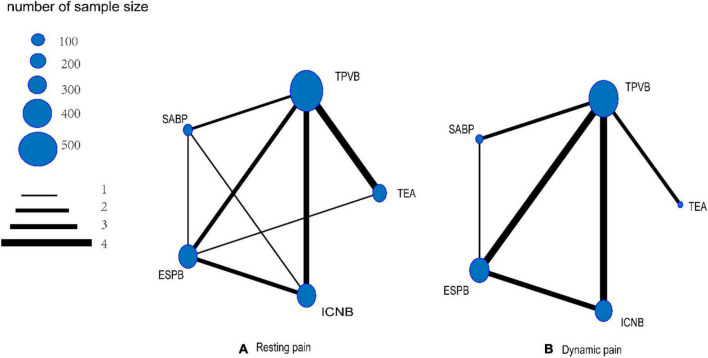
Network geometry.

Visual network geometry (Figures 2A,B) was used to display each arm. Each treatment is represented by a unique node whose size relies on the number of samples the treatment contributes to the network. Regarding the analgesic method used for resting pain (Figure 2A), five comparisons among locoregional analgesia groups were described in our NMA. TPVB was the most frequent intervention and was investigated in 10 arms (*n* = 510), and the next most common interventions were TEA, involving 6 arms (*n* = 194), ICNB, involving 6 arms (*n* = 162), ESBP, involving 5 arms (*n* = 162), and SABP, involving 4 arms (*n* = 121). Regarding the analgesic method used for dynamic pain (Figure 2A), TPVB was the most frequent intervention and was investigated in 10 arms (*n* = 352), and the next most common interventions were ESBP, involving 5 arms (*n* = 138), ICNB, involving 5 arms (*n* = 135), SABP, involving 3 arms (*n* = 91), and TEA, involving 2 arms (*n* = 65).

**FIGURE 3:**
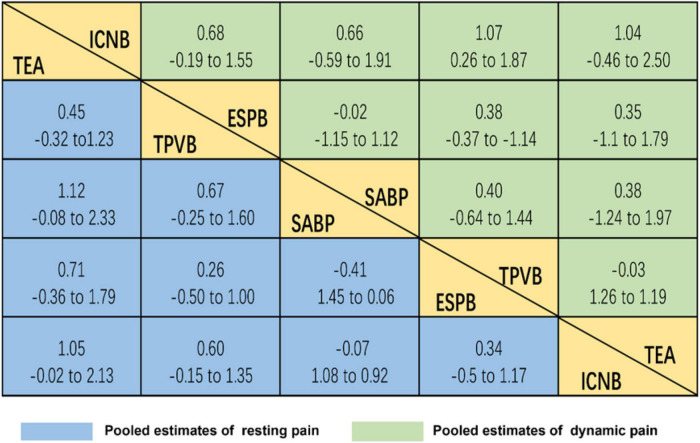
Relative effect sizes of efficacy at post-treatment according to network meta-analysis. Treatments are orders in the rank of their chance of being the best treatment. For efficacy in post-treatment, standardized mean differences (SMDs) more than 0 favor the column-defining treatment. Significant superiority of locoregional analgesia for pain management after VATS.

Considering the efficacy of each intervention from baseline to completion, 5 therapies were significantly superior to locoregional analgesia for pain management after VATS (Figure 3), and the estimates of the underlying effect varied relatively widely. TEA was the best option for resting pain [SMD (standard mean difference) = 1.12, CrI (credible interval): (–0.08–2.33)], followed by TPVB [SMD = 0.67, CrI: (−0.25 to 1.60)]. Regarding analgesic methods for dynamic pain, TPVB was the best [SMD = 1.07, CrI: (0.26–1.87)], followed by TEA [SMD = 1.04, CrI: (−0.46 to 2.50)].

**FIGURE 4:**
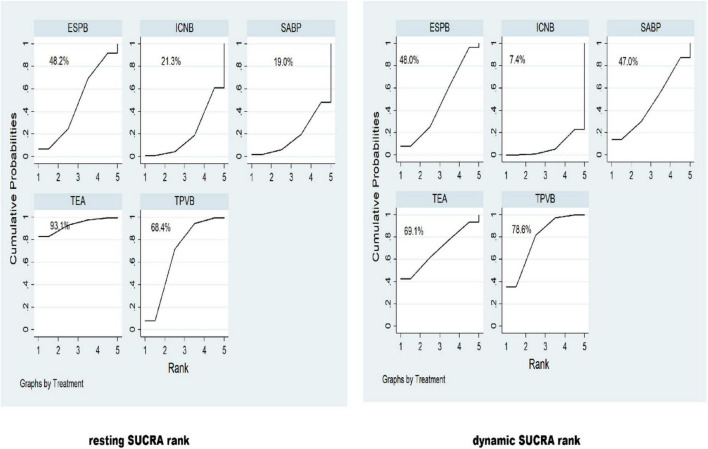
The surface under the cumulative ranking curve (SUCRA) was presented as a simple numerical statistic, with cumulative ranking probability plots summarized for each treatment. A SUCRA with a higher value denotes a greater likelihood of a given treatment being in the top rank or highly effective, while zero indicates that the treatment is certain to be the worst. A surface under the cumulative ranking curve (SUCRA) line was drawn to rank locoregional analgesia for pain management.

A SUCRA line was drawn to rank locoregional analgesia for pain management (shown in Figure 4), and it indicated that TEA (resting SUCRA = 93.1%) (dynamic SUCRA = 69.1%) and TPVB (resting SUCRA = 78.6%) (dynamic SUCRA = 68.4%) still had the greatest postoperative analgesia effect after VATS. This approach enabled a legitimate comparison of the abovementioned psychosocial treatments since there was no statistically significant discrepancy between the direct and indirect estimates investigated using the node-splitting method (TEA vs. TPVB *p* value = 0.548, TEA vs. ESPB *p* value = 0.552, TPVB vs. SABP *p* value = 0.044, TPVB vs. ESPB *p* value = 0.961, TPVB vs. ICNB *p* value = 0.026, SABP vs. ESPB *p* value = 0.654, SABP vs. ICNB *p* value = 0.077, ESPB vs. ICNB *p* value = 0.212).

## Discussion

Various regional pain blocks can help with acute and chronic pain after VATS. In this NMA, we reviewed locoregional analgesia for pain management following VATS and determined which strategies provide the best pain relief. The results suggest that TEA and TPVB are the optimal analgesia interventions for VATS, and the above evidence was reinforced by a previous meta-analysis (47). When patients cannot undergo TEA or TPVB due to side effects such as hypotension, urine retention, nausea, or vomiting (11), ESPB could be administered as an alternate analgesic. This view is similar to that of Cassai (48). Compared to no analgesia, these locoregional analgesia methods demonstrated excellent analgesic effects following thoracoscopic surgery, although these findings require further study.

Because no locoregional analgesia method has been identified as the best therapy, a reliable treatment must be adopted. TEA and TPVB had a considerable analgesic effect when compared to these locoregional analgesics. Our results complement an earlier study (47) that shows consistency between the efficacy of TEA and TPVB in improving postoperative chest pain. A recent survey comparing the analgesic efficacy and side, effects of epidural vs. PVB for VATS showed that PVB had a better short-term side effect profile, including fewer major postoperative complications and fewer unexpected Intensive Care Unit admissions (49). The benefits seen with PVB can be explained by the blocking of unilateral intercostal nerves only, with preservation of respiratory and sympathetic function on the contralateral side (50). To provide a more complete picture, we looked not only at static pain relief in patients with regional block but also at dynamic pain relief, particularly with TPVB. TPVB inhibits stress and inflammation without producing significant hemodynamic changes (51). TPVB maintained cellular immunity better than other forms of local anesthesia. The use of effective regional block analgesia may help patients cope with dynamic pain, return their lungs to normal function sooner after surgery and help them recover more quickly overall (52).

Moreover, in light of our positive findings, and different from Federico’s view (20), we verified that ESBP has a very good impact following thoracoscopic surgery. ESPB is an emerging technique that has been applied in a wide variety of fields, and notably, even beginners can easily learn the technique (53). The physical distribution of local anesthetics to the thoracic paravertebral region and related brain tissues is the most compelling theory (54). ESPB conducted at the level of the T5 transverse process was capable of delivering significant thoracic analgesia, ranging from T3 to T9 throughout the hemithorax, and was predicted to extend to the paravertebral region, acting on both the ventral and dorsal rami of the spinal neurons (55). Diffusion into the paravertebral space through the intertransverse connective tissue complex may persist for an extended period of time. Hence, ESPB should have a comparable impact to TPVB if the anterior spreading to the thoracic paravertebral space is adequate, it may be a straightforward replacement to PVB.

SABP and ICNB also have analgesic properties after thoracoscopic surgery. SABP is simple to use and provides postoperative dynamic pain relief. SAPB is conducted by injecting a specific concentration and volume of local anesthetic between the serratus anterior and intercostal muscles and blocking the lateral cutaneous branches of the T2–T9 spinal neurons (56). Only SAPB was shown to be effective in blocking the long thoracic nerve, which regulates pain caused by injury to the serratus muscle and strain on surrounding tissues. The long thoracic nerve, as a motor nerve, is engaged in afferent nociception *via* sensory innervation and connection (57). As a result, SABP is effective in reducing postoperative discomfort produced by respiratory movement and movement pulling. ICNB is a well-known conventional treatment for pain management following thoracic surgery. It has a comparable analgesic effect to SABP, but it involves segmental localized analgesia, requiring numerous injections at different sites to increase the analgesic impact (58). However, in terms of analgesia, SABP and ICNB were placed lower in this NMA. Because SABP and ICNB are not known to treat visceral pain, interindividual differences in efficacy are possible, given that injection site and volume influence the degree of diffusion and the mass of local anesthetic reaching target nerves. Finally, the number of studies covered was limited, with only four SABP and six ICNB investigations, and more research is needed to establish their usefulness after VAST.

Future studies should take into account a number of other variables as well. The concept of extended release has been recently introduced. Bupivacaine liposomes had a 72-h duration of action, which just rekindles the interest in the use of single-shot anesthetics and prolongs relief of thoracoscopic postoperative pain. Improving pain control during the perioperative phase will not only reduce LOS and patient satisfaction, but it will also allow for a reduction in opioid use (59, 60). Meanwhile, it eliminates the need for ongoing nerve blocks, makes catheterization easier, and increases the risk of infection (50). Chest tubes must be used (for a varying number of times) during the operation (61). Post-VATS discomfort has been linked to chest tubes; therefore, the number, size and location of chest tubes and the duration of chest tube use should be tracked and reported (62). Then, studies can focus on minimizing postoperative chest tube discomfort and determining whether combined regional block analgesia can help with thoracic catheter implantation discomfort. The reduction of CPSP after surgery is also a matter of concern; the combined use of postoperative sedation analgesia and regional block analgesia may prevent chronic inflammation and the development of CPSP.

### Strengths and Limitations

Our NMA was the first to evaluate each treatment separately and compare the most common locoregional analgesia methods of pain management after VATS, rather than just categorizing therapies into TEA or TPVB. Furthermore, with the rise in postoperative thoracoscopic regional block studies in recent years, it is more important than ever to efficiently organize and evaluate these studies so that the best postoperative thoracoscopic analgesia treatments can be provided.

It is also necessary to recognize the study’s shortcomings. First, because so many regional block analgesia methods have not been included in RCTs for different reasons, information on the efficacy of analgesia and treatments is limited, making it difficult to draw definite conclusions from our NMA. Although the current study included patients who underwent VATS, the medication concentrations and technical aspects were not uniform, which may have led to some heterogeneity. Finally, we could not rely on the time points at which the pain ratings were collected; therefore, we omitted them. We compared values from 24 h after surgery to eliminate any potential for bias.

## Conclusion

Our NMA concluded that interoperative TEA, TPVB, and ESPB are the best treatments for VATS when used simultaneously. ESPB also has a strong analgesic effect. These results may improve existing locoregional analgesia standards and future postoperative analgesia trial designs. Additional research is required to identify the best method of regional analgesia for pain management post-VATS.

## Author Contributions

JL, YL, and CG conducted the database search, screened and extracted data for the manuscript, and had primary responsibility in writing this article. JL, LY, FG, and JY interpreted the data, drafted the initial manuscript, and contributed to the discussion and editing. XZ, JC, XC, and TZ supervised data collection and critically edited the final manuscript. All authors approved the final manuscript as submitted, agreed to be accountable for all aspects of the work, and read and approved the final manuscript.

## Conflict of Interest

The authors declare that the research was conducted in the absence of any commercial or financial relationships that could be construed as a potential conflict of interest.

## Publisher’s Note

All claims expressed in this article are solely those of the authors and do not necessarily represent those of their affiliated organizations, or those of the publisher, the editors and the reviewers. Any product that may be evaluated in this article, or claim that may be made by its manufacturer, is not guaranteed or endorsed by the publisher.
