# Supplementary material for: Regional Analgesia in Video-Assisted Thoracic Surgery: A Bayesian Network Meta-Analysis
Source: Front Med (Lausanne). 2022 Apr 6;9:842332. doi: 10.3389/fmed.2022.842332 (PMC9019113; doi:10.3389/fmed.2022.842332)
Supplement: Supplementary file 1 [file Data_Sheet_1.pdf]

Supplementary materials

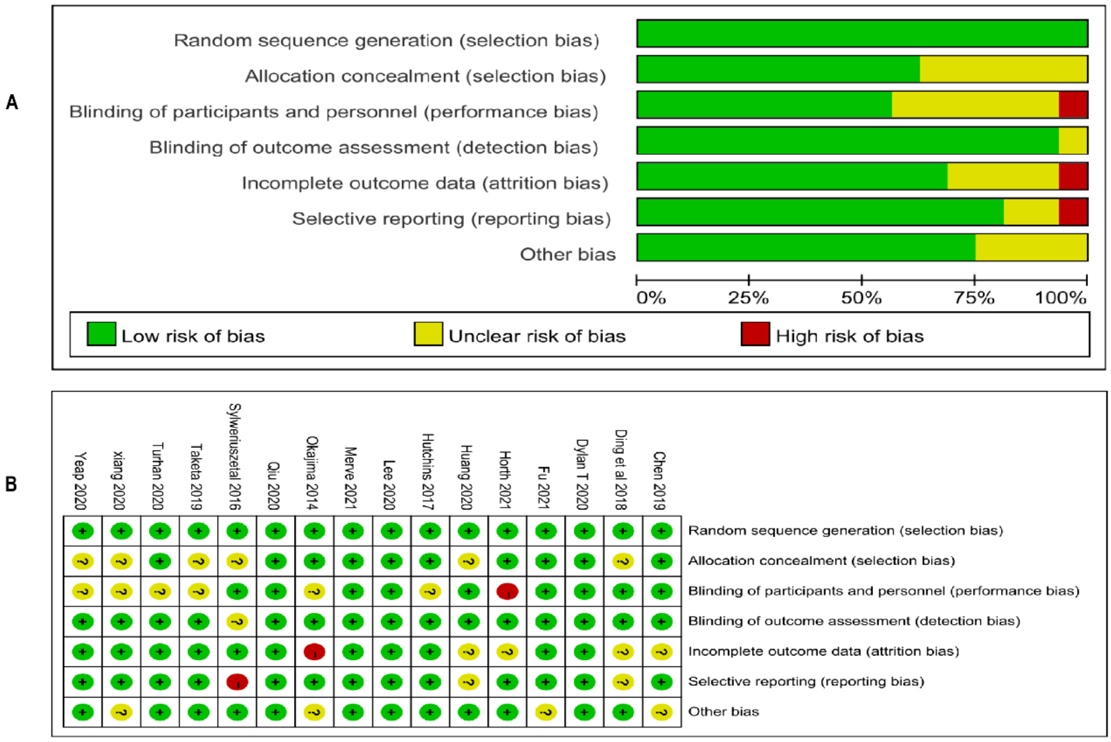

A: risk of bias graph B: risk of bias summary

Figure S1 study-level quality

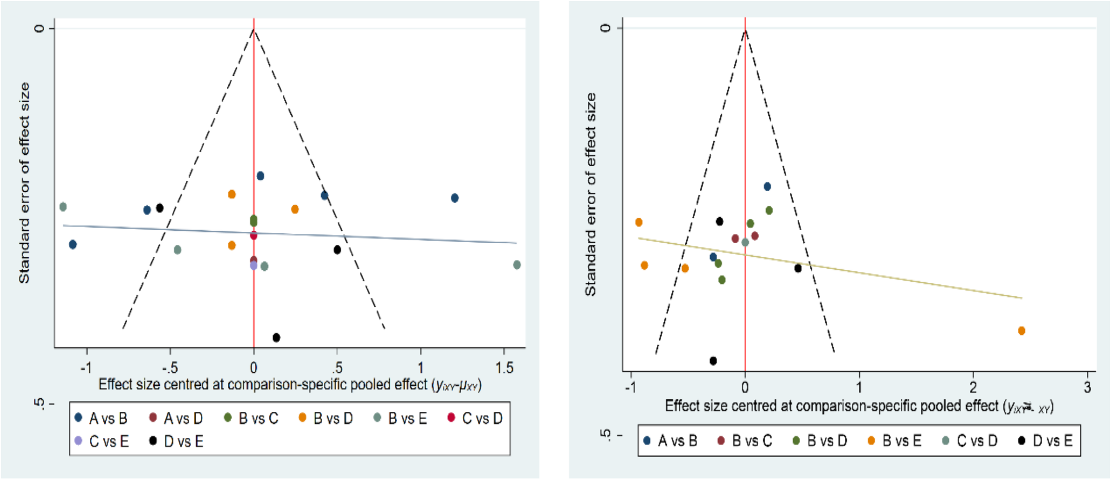

A: Resting Pain *B: Dynamic Pain*

A: TEA epidural block; B: TPVB paravertebral block; C: SABP serratus anterior plane block; D: ESPB erector spinae plane; E: ICNB intercostal nerve block.

Figure S2 funnel plot
